# Journey to the east: Diverse routes and variable flowering times for wheat and barley *en route* to prehistoric China

**DOI:** 10.1371/journal.pone.0187405

**Published:** 2017-11-02

**Authors:** Xinyi Liu, Diane L. Lister, Zhijun Zhao, Cameron A. Petrie, Xiongsheng Zeng, Penelope J. Jones, Richard A. Staff, Anil K. Pokharia, Jennifer Bates, Ravindra N. Singh, Steven A. Weber, Giedre Motuzaite Matuzeviciute, Guanghui Dong, Haiming Li, Hongliang Lü, Hongen Jiang, Jianxin Wang, Jian Ma, Duo Tian, Guiyun Jin, Liping Zhou, Xiaohong Wu, Martin K. Jones

**Affiliations:** 1 Department of Anthropology, Washington University in St. Louis, St. Louis, MO, United States of America; 2 McDonald Institute for Archaeological Research, University of Cambridge, Cambridge, United Kingdom; 3 Institutue of Archaeology, Chinese Academy of Social Sciences, Beijing, China; 4 Institute for the History of Natural Sciences, Chinese Academy of Sciences, Beijing, China; 5 Research Laboratory for Archaeology and the History of Art, University of Oxford, Oxford, United Kingdom; 6 Scottish Universities Environmental Research Centre (SUERC), University of Glasgow, East Kibride, United Kingdom; 7 Birbal Sahni Institute of Palaeobotany, Lucknow, India; 8 Department of AIHC and Archaeology, Banaras Hindu University, Varnasi, India; 9 Department of Anthropology, Washington State University, Vancouver, WA, United States of America; 10 Department of Archaeology, Vilnius University, Vilnius, Lithuania; 11 Key Laboratory of Western China’s Environmental Systems, Lanzhou University, Lanzhou, China; 12 Department of Archaeology, Sichuan University, Chengdu, China; 13 Department of History of Science and Archaeometry, University of Chinese Academy of Sciences, Beijing, China; 14 School of Cultural Heritage, Northwest University, Xi’an, China; 15 School of History and Culture, Shandong University, Jinan, China; 16 Laboratory for Earth Surface Processes, Peking University, Beijing, China; 17 School of Archaeology and Museology, Peking University, Beijing, China; National Cheng Kung University, TAIWAN

## Abstract

Today, farmers in many regions of eastern Asia sow their barley grains in the spring and harvest them in the autumn of the same year (spring barley). However, when it was first domesticated in southwest Asia, barley was grown between the autumn and subsequent spring (winter barley), to complete their life cycles before the summer drought. The question of when the eastern barley shifted from the original winter habit to flexible growing schedules is of significance in terms of understanding its spread. This article investigates when barley cultivation dispersed from southwest Asia to regions of eastern Asia and how the eastern spring barley evolved in this context. We report 70 new radiocarbon measurements obtained directly from barley grains recovered from archaeological sites in eastern Eurasia. Our results indicate that the eastern dispersals of wheat and barley were distinct in both space and time. We infer that barley had been cultivated in a range of markedly contrasting environments by the second millennium BC. In this context, we consider the distribution of known haplotypes of a flowering-time gene in barley, *Ppd-H1*, and infer that the distributions of those haplotypes may reflect the early dispersal of barley. These patterns of dispersal resonate with the second and first millennia BC textual records documenting sowing and harvesting times for barley in central/eastern China.

## Introduction

### The eastward dispersals of wheat and barley

Wheat and barley were domesticated in the Fertile Crescent as winter crops, as were other southwest Asian crops. By *c*. 500 BC, the geographical distribution of the Fertile Crescent crops, free-threshing wheat (*Triticum aestivum*) and naked barley (*Hordeum vulgare* ssp. *vulgare*), stretched from the Atlantic to the Pacific, north to Scandinavia, and south to the Indian Ocean. Within this vast geographical span, barley is notable for its successful cultivation in altitudinal and latitudinal extremes. Today, it is commonly cultivated in regions such as Scandinavia (high latitude) and the northern Tibetan Plateau (mid-latitude but high altitude). This prompts the question: when did ancient farmers alter the seasonality of barley’s life cycle to cultivate it as a summer crop? The westward dispersal of barley to higher latitudinal Europe associated with shifting growth seasonality has been reasonably established [1]. In this paper, we examine the eastward dispersal of barley across high altitudinal regions, and consider the contrast between the pattern for barley and that for wheat, in the context of its distinct ecology and adaptive responsiveness, specifically in relation to seasonality and flowering time.

In the context of a growing scholarly interest in the early food globalisation [2,3] and the drivers underling the adaptation of exotic grains into existing agricultural system [4–7], the chronology and routes of the westward expansion of the ‘Neolithic founder crops’ from southwest Asia to Europe have been much discussed [8–10], and recent research has sought to elucidate the eastward expansion of these crops to India and China [2,11–17]. Evidence for the eastward expansion of the Fertile Crescent crops includes archaeobotanical data showing the cultivation/domestication of various hulled and free-threshing wheat and barley varieties in southwest Asia from at least 8,000 BC [18], both hulled and free-threshing wheat and barley in Turkmenistan from around 6,500–3,000 BC [19,20] and in Pakistan from around 6,000–3,000 BC [21–25]. After these initial records, hulled and free-threshing/naked wheat and barley spread further east towards India during the third millennium BC [25–28]. In the north and along the ‘Inner Asian Mountain Corridor’ [29], further expansion appears to be restricted to free-threshing/naked forms, with sites in Afghanistan, Tajikistan, Kazakhstan and Kyrgyzstan reporting free-threshing wheat and naked barley in the third and second millennia BC [14,16,30–33]. Further dispersals brought wheat and barley cultivation into south India and China.

Drawing upon recent evidence from directly dated wheat grains, separate dispersal routes may be distinguished for wheat along the north and south of the Tibetan Plateau, moving into China and India respectively (see Figure A and the additional text in S1 File) [11].

### Environmental challenges and the genetic control of flowering time

To complete their life cycles, the time of flowering of plants needs to coincide with favourable weather conditions, in order to avoid sensitive floral tissues being damaged through extremes of temperature or drought [1,34]. In their regions of origin, wheat and barley need to complete their life cycles before the summer drought arrives. This is achieved by flowering being induced by increasing day-lengths as spring/summer approaches. When these southwest Asian crops spread to novel latitudes and altitudes such a seasonal response may prove maladaptive. How this applies to cereal cultivation depends upon the taxon; the two principal cereals moving east had different ecological attributes, and varying potential pathways of spread. In contrast to the relatively demanding taxon, wheat, barley has a notably wide ecological range, manifest in its successful cultivation at altitudinal and latitudinal extremes [35].

Cultivation at extremes of latitude and altitude leads to selection pressure upon the plant’s seasonal response genes. In barley, these genes include *Photoperiod-H1* (*Ppd-H1*) [36,37]. Mutations at the *Ppd-H1* gene locus have been shown to result in the switching off of the photoperiod response, enabling growth under different patterns of seasonality. The severe winter frosts and snow of northerly latitudes and high altitudes favour the ‘spring growth habit’ of spring-sown crops. The acquisition of a spring growth habit and photoperiod insensitivity in barley is thought to be key to its adaptation to the high latitudes in northern Europe [38] and the high altitudes of the Tibetan Plateau [39–41]. The phylogeographic analysis of the *Ppd-H1* gene in wild and landrace barley has elucidated patterns of early agricultural dispersal across Europe [38,42]. Similar analyses have been conducted in relation to the eastward dispersal of barley across Asia [1].

## Materials and methods

### Archaeobotanical materials and radiocarbon analyses

**Fig 1:**
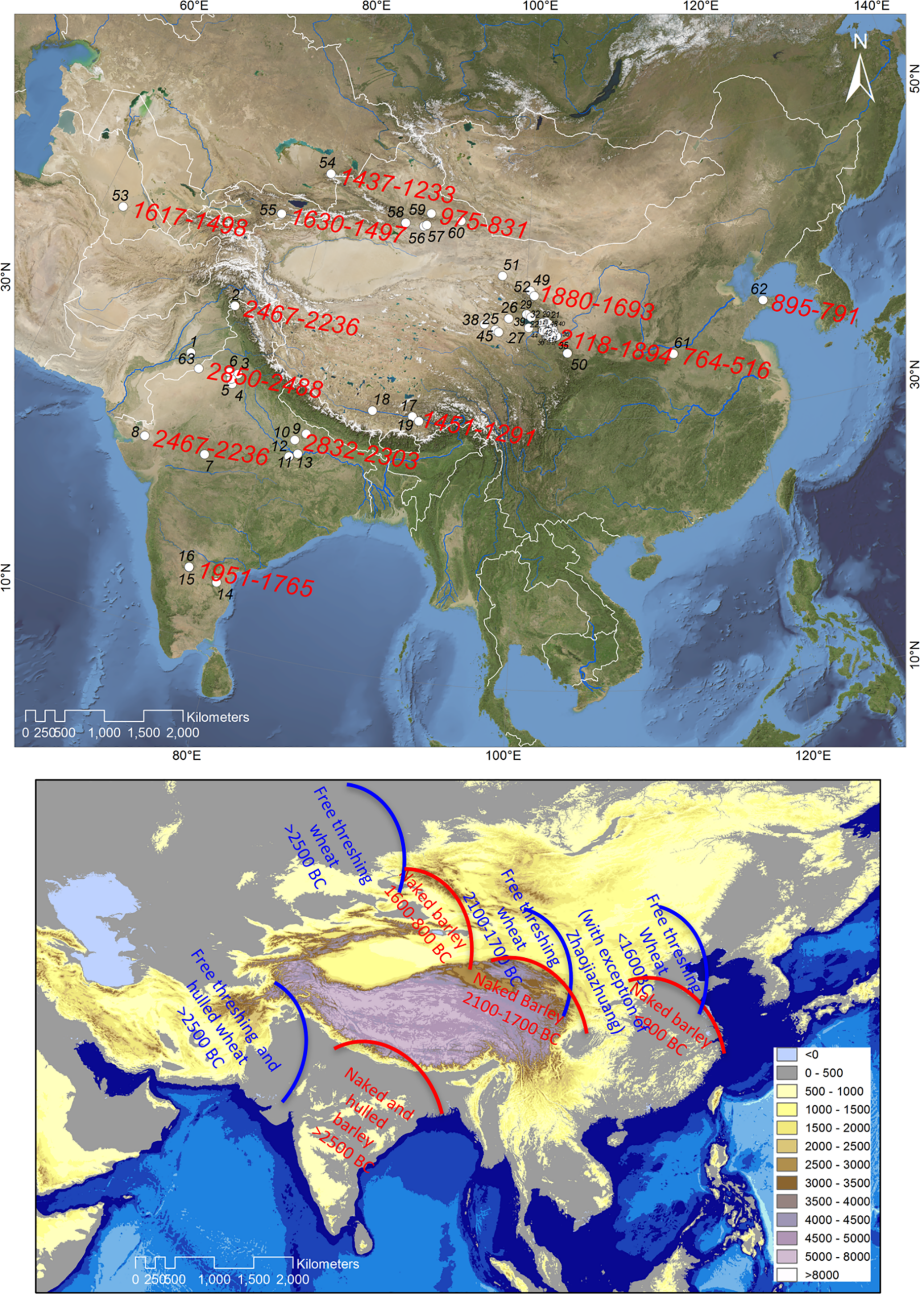
Sites reporting direct radiocarbon measurements of barley grains. The oldest individually dated grains of barley from each region are indicated. The pathways to the east for wheat and barley are probably distinct from each other. The introduction of wheat and barley into South Asia involves both hulled and naked forms, and a millennium older than the introduction of wheat and barley into East Asia, which were restricted to naked forms. Free-threshing wheats spread to China with a route to the north of the Tibetan Plateau. Naked barley is likely to have been introduced to China via southern highland routes that remain to be identified. 1. Harappa, 2. Kanispur, 3. Balu, 4. Tigrana, 5. Masudspur VII, 6. Burj, 7. Khirsara, 8. Kanmer, 9. Lahuredewa, 10. Damdama, 11. Agaibir, 12. Mahagara, 13. Koldihwa, 14. Hanumantaraopeta, 15. Sannarachamma, 16. Hiregudda, 17. Changguogou, 18. Khog Gzung, 19. Banga, 20. Changning, 21. Fengtai, 22. Xiasunjiazhai, 23. Gongshijia, 24. Jiaoridang, 25. Tawendaliha, 26. Hongshanzuinanpo, 27. Qiezha, 28. Huidui, 29. Lagalamaerma, 30. Luowalinchang, 31. Dongfengxinan, 32. Kalashishuwan, 33. Weijiabao, 34. Tuanjie, 35. Erfang, 36. Wenjia, 37. Bayan, 38. Talitaliha, 39. Caodalianhuxi, 40. Shuangerdongping, 41. Yingpandi, 42. Xiawatai, 43. Lalongwa, 44. Gagai, 45. Keer, 46. Lamuzui, 47. Yanguo, 48. Shawuang, 49. Heishuiguo, 50. Mogou, 51. Huoshaogou, 52. Donghuishan, 53. Ojakly, 54. Tasbas, 55. Aigyrzhal-2, 56. Yanghai, 57. Shengjindian, 58. Yuergou, 59. Sidaogou, 60. Shirenzigou, 61. Wangchenggang, 62. Zhaogezhuang, 63. 4-MSR.

Seventy carbonised grains of barley from China (n = 54), India (n = 12), Kyrgyzstan (n = 1) and Pakistan (n = 3) were selected for radiocarbon (^14^C) analyses at several laboratories, including Oxford Radiocarbon Accelerator Unit (OxA), the Laboratory of Earth Surface Processes (QAS) and Radiocarbon Accelerator Laboratory (BA), Peking University, Beta-Analytic (Beta) and Direct AMS (D-AMS). The sample preparation methods undertaken at these laboratories were similar, with a standard acid-base-acid (ABA) chemical pre-treatment method followed by combustion and graphitisation prior to accelerator mass spectrometry (AMS) [43]. These new radiocarbon determinations were subsequently collated with previously published data, with the summary data for these samples, as well as for the new samples selected herein, presented in Table 1 and Fig 1.

**Table 1:** Direct radiocarbon dates for archaeobotanical barley grains from East, Central and South Asia. Data include radiocarbon determinations carried out in this study and those that have been previously published. The radiocarbon data have been calibrated using the IntCal13 calibration curve, and are presented at the 95.4% probability range.

Region	Site	Radiocarbon Lab no.	Conventional ^14^C age BP (±1σ)	Calibrated age (cal. BC)	
Kashmir, India (NW India/Indus)	Kanispur	Beta-427232	3880±30	2467–2236	this study
Haryana, India	Balu	Beta-427233	3990±30	2575–2466	this study
(NW India/Indus)	Tigrana	OxA-29982	3981±36	2581–2349	this study
		OxA-30017	3907±27	2471–2299	this study
	Masudspur VII	Beta-427238	4040±30	2832–2474	this study
		Beta-427239	3980±30	2578–2457	this study
	Burj	OxA-26476	3981±36	2581–2349	this study
Rajasthan, India	4-MSR	GdA-4806	4065±30	2850–2488	this study
(NW India/Indus)		GdA-4807	4045±30	2834–2475	this study
Punjiab, Pakistan	Harappa	OxA-30062	3443±27	1879–1683	this study
(NW India/Indus)		OxA-30063	3446±29	1879–1686	this study
		OxA-30064	3463±28	1881–1693	this study
Gujarat, India	Khirsara	Beta-427231	3750±30	2281–2038	this study
(NW India/Indus)	Kanmer	PLD16352	3880±30	2467–2236	[52]
		PLD17147	3835±20	2431–2202	[52]
Uttar Pradesh,	Lahuredewa	Erl-6903	3827±147	2850–1884	[53]
India (Ganges)	Damdama	DAMMESO-1	3984±54	2832–2303	[53]
	Agaibir	D-AMS018161	2866±31	1126–927	this study
		D-AMS018163	2807±33	1049–851	this study
	Mahagara	OxA-14097	2546±29	801–550	[54]
	Koldihwa	OxA-14094	3269±29	1621–1461	[54]
Karnataka, India	Hanumantaraopeta	BA04394	3295±30	1639–1502	[55]
(S India)	Sannarachamma	BA05776	3125±40	1496–1284	[55]
		R 28680/6	3361±40	1746–1532	[55]
		R 28680/3	3536±30	1951–1765	[55]
	Hiregudda	R 28680/17	3382±35	1766–1564	[55]
Tibet, China	Khog Gzung	BA140576	2970±20	1260–1121	this study
(Tibetan Plateau)		BA140577	2930±20	1211–1052	this study
		BA140578	3040±25	1393–1211	this study
	Bangtangbu	Beta-450799	2960±30	1263–1056	this study
	Bangga	Beta-448782	2590±30	820–595	this study
Qinghai, China	Changning	QAS1318	3585±25	2021–1884	this study
(Tibetan Plateau)		QAS1319	3570±20	2010–1881	this study
	Fengtai	QAS1322	2620±20	818–789	this study
	Xiasunjiazhai	BA120205	3665±25	2136–1959	[40]/this study
	Gongshijia	Beta-303689	3620±30	2118–1894	[40]/this study
	Jiaoridang	BA110890	3190±30	1514–1412	[40]/this study
	Gongshijia	BA110893	3165±35	1508–1318	[40]/this study
	Tawendaliha	Beta-324460	3110±30	1437–1288	[40]/this study
	Hongshanzuinanpo	BA120203	3075±30	1417–1261	[40]/this study
	Qiezha	Beta-353860	3070±30	1415–1236	[40]/this study
	Huidui	BA120198	3060±35	1412–1228	[40]/this study
	Lagalamaerma	Beta-324457	3060±30	1411–1231	[40]/this study
	Louwalinchang	BA110895	3055±40	1417–1213	[40]/this study
		Beta-303691	3050±30	1401–1226	[40]/this study
	Dongfengxinan	Beta-292121	3010±40	1392–1123	[40]/this study
	Kalashishuwan	BA120194	3020±25	1388–1134	[40]/this study
	Weijiabao	BA120184	2905±30	1207–1008	[40]/this study
	Tuanjie	BA110892	2930±35	1226–1014	[40]/this study
	Erfang	Beta-303688	2910±30	1209–1011	[40]/this study
	Wenjia	BA110888	2890±30	1195–978	[40]/this study
	Bayan	BA120192	2860±20	1111–941	[40]/this study
	Talitalliha	BA120176	2840±30	1108–917	[40]/this study
		Beta-324459	2770±30	997–839	[40]/this study
	Caodalianhuxi	Beta-344749	2830±30	1083–906	[40]/this study
	Shuangerdongping	BA110903	2770±25	994–840	[40]/this study
	Yingpandi	BA120200	2760±25	976–832	[40]/this study
	Xiawatai	BA120183	2750±30	976–822	[40]/this study
	Lalongwa	BA110894	2685±30	899–803	[40]/this study
	Gagai	BA110900	2550±30	801–551	[40]/this study
	Keer	BA120178	2550±30	801–551	[40]/this study
	Lamuzui	Beta-292120	2520±40	798–521	[40]/this study
	Yangou	BA110891	2460±30	758–429	[40]/this study
	Shawuang	BA120193	2325±30	481–257	[40]/this study
Gansu, China	Heishuiguo	QAS1312	3460±25	1880–1693	this study
		QAS1313	3360±25	1739–1565	this study
		QAS1315	3355±30	1740–1535	this study
		QAS1317	3400±25	1750–1630	this study
	Mogou	Beta-427234	3330±30	1689–1528	this study
	Huoshaogou	Beta-427235	2330±30	486–262	this study
	Donghuishan	BA06022	3235±35	1611–1434	[13]
		BA06028	3175±35	1518–1324	[13]
		BA06026	3235±35	1611–1434	[13]
		BA06032	3280±38	1643–1454	[13]
		Beta-427236	3150±30	1500–1311	this study
Kazakhstan	Tasbas 2a	OS92277	3090±40	1437–1233	[16]
(C Asia)		OS91990	3030±35	1405–1132	[16]
Turkmenistan (C Asia)	Ojakly	OS92543	3270±25	1617–1498	[16]
Kyrgyzstan (C Asia)	Aigyrzhal-2	Beta-435511	3280±30	1630–1497	this study
Xinjiang, China	Sidaogou	BA111398	2757±25	975–831	this study
(C Asia)		BA111399	2470±20	764–491	this study
		BA111401	2535±25	796–549	this study
	Shirenzigou	Beta-435992	2150±30	356–61	this study
	Yanghai	Beta-440290	2430±30	750–405	this study
	Yuergou	Beta-440292	2170±30	360–116	this study
	Shengjindian	Beta-440291	2100±30	198–47	this study
Henan, China (C/E China)	Wangchenggang	QAS1306	2475±20	764–516	this study
Shandong, China (C/E China)	Zhaogezhuang	Beta-427237	2650±30	895–791	this study

We aimed to incorporate as many recently recovered barley grains from sites in East, South and Central Asia as possible. One of the recent advances in archaeological research that has enabled the recovery of these grains is the application of flotation technology in these regions. Flotation has thus been applied at several hundred sites in China alone in the past decade [44], many of which report the presence of southwest Asian crops. Archaeobotanical analyses were undertaken in several institutions, including archaeobotanical laboratories at the Institute of Archaeology, Chinese Academy of Social Sciences, University of Chinese Academy of Sciences, Shandong University, Sichuan University, the Archaeobotany Laboratory at Birbal Sahni Institute of Palaeosciences, and the George Pitt-Rivers Laboratory, University of Cambridge. Barley grains that were recovered from deposits thought to date from the third and second millennium BC–i.e. relating to the earliest appearance of barley across these regions–were selected for radiocarbon analyses. The data reported here all relate to ‘direct’ radiocarbon determinations upon individual barley grains themselves, rather than dating of material from the associated archaeological contexts, and therefore provide wholly reliable chronological information regarding the presence of barley at these sites.

### Chronological modelling

**Fig 2:**
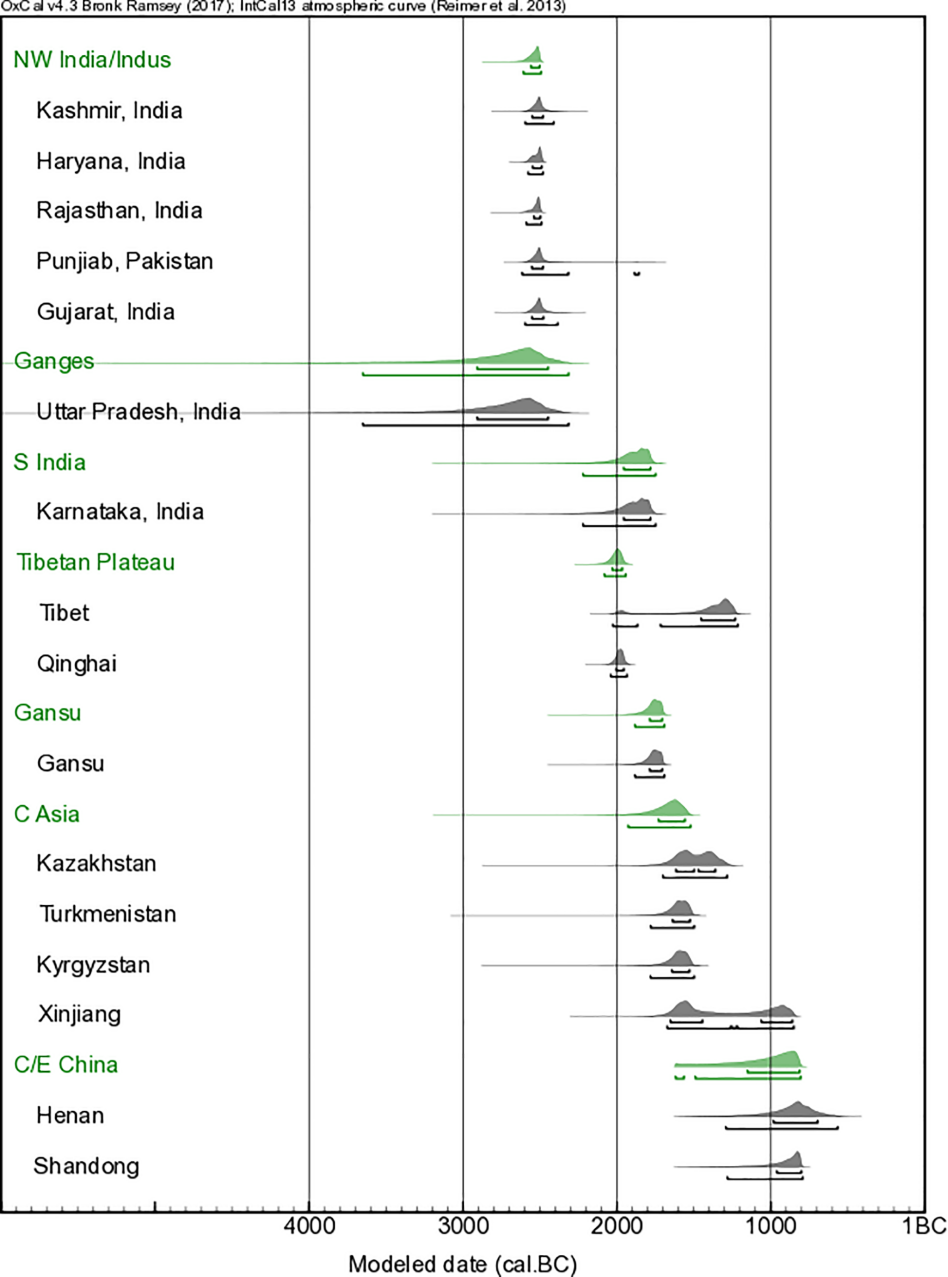
The ‘first appearance dates’ of barley derived by Bayesian statistical modeling for fifteen regional groupings (further grouped into seven broader regional groupings) of archaeological sites across central, south and east Asia. (See Supporting Information for full details regarding the model construction). The horizontal bars below each of the probability density functions reflect the 68.2% and 95.4% highest probability density ranges, respectively. These results show a north to south chronological sequence of the first appearance dates of barley within South Asia, and a south to north sequence between South and East Asia.

**Fig 3:**
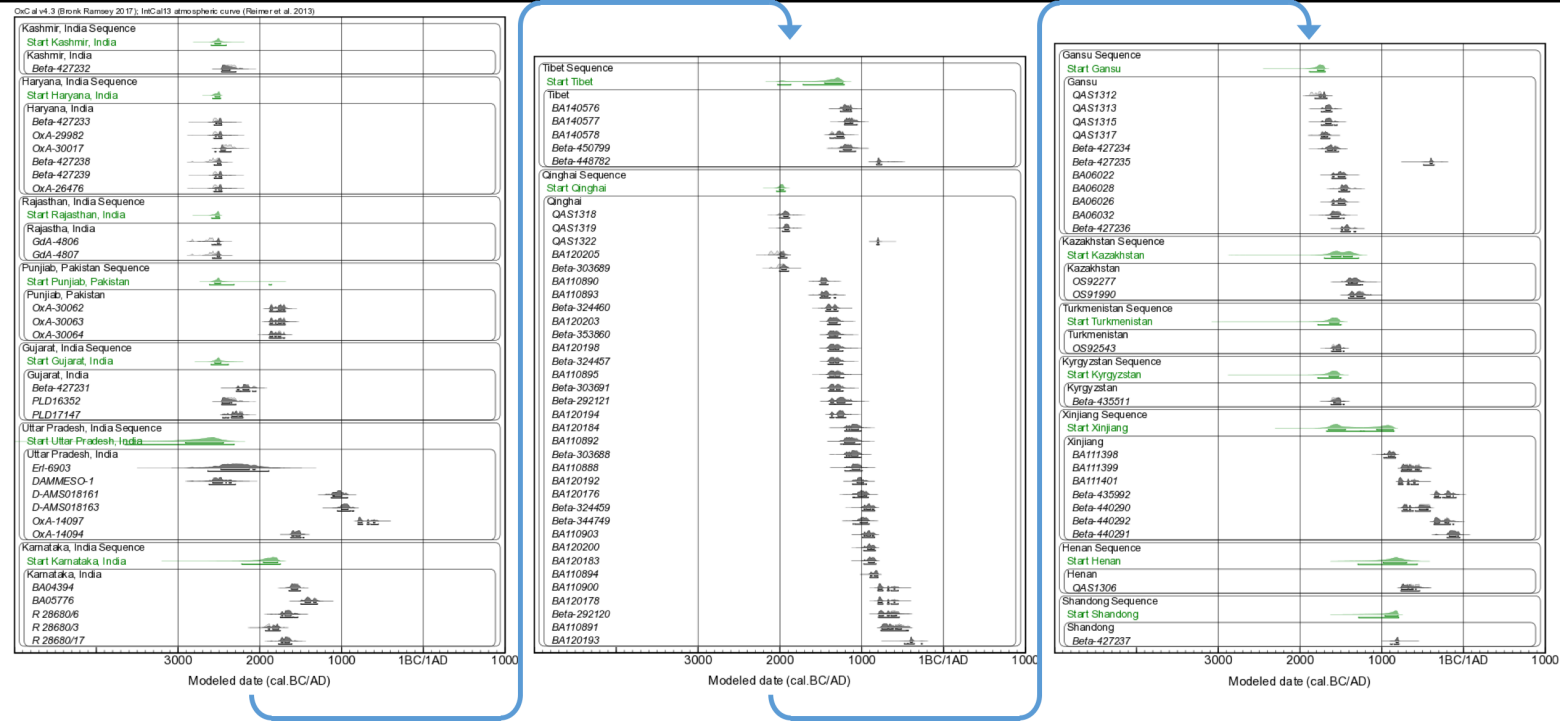
The implied ‘first appearance dates’ (i.e. ‘Start’ *Boundaries*) of the fifteen regions derived from the Bayesian statistical model (green). The contributing radiocarbon data are additionally plotted (with modeled data in darker gray overlying the unmodeled, calibrated data in lighter gray). The horizontal bars below each of the probability density functions reflect the 68.2% and 95.4% highest probability density ranges, respectively.

In order to provide more refined estimates of the ‘first appearance dates’ of barley within each region, we undertook Bayesian statistical modeling of the collated dataset using the freely available OxCal ver. 4.3 software [45,46], and applying the IntCal13 radiocarbon calibration curve [47]. These results are presented in Figs 2 and 3. All radiocarbon determinations were divided into a series of independent *Phases*, representing the fifteen geographical regions identified in Table 1. These sixteen *Phases* were unrelated to each other; i.e. there were no assumptions, *a priori*, as to the relative ordering of the respective *Phases*. A combination of *Boundaries* and *Tau_Boundaries* were applied at the ‘Start’ and ‘End’ of each of the fifteen model *Phases*, respectively. This combination of *Boundaries* and *Tau_Boundaries* provides an exponentially decreasing *Phase*, allowing for the bias towards older samples within each *Phase* that results from our research focus on providing the earliest dating barley grains from each site. The Start *Boundaries* of each *Phase* thus provide the model estimated ‘first appearance’ date from each of the fifteen regions, and generally pre-date the earliest individual radiocarbon dated samples from each region slightly (see Fig 3). A second, parallel series of seven *Phases* representing broader geographical scale regions from which the fifteen more localised geographical regions were located (namely: Northwest India/Indus, Ganges, South India, Tibetan Plateau, Gansu, Central Asia, and Central/Eastern China) was run within the same OxCal model (Fig 2). We group Kashmir with sites from the broader Indus region and northwest India. It should be noted that Kashmir is normally regarded as north India. By grouping more radiocarbon dated samples within these broader regional *Phases*, the model could produce more precise Start *Boundaries*–i.e. more precise ‘first appearance dates’–which allow for more rigorous archaeological interpretation.

## Results

We have here collated published radiocarbon dates for early barley finds, and augmented that evidence by directly dating 70 barley grains (Table 1 and Fig 1). Bayesian statistical modeling for the first appearance dates of barley within each region has been employed and the results are shown in Figs 2 and 3. These results are considered together with recently published dates for wheat [11]. Where chronologically appropriate, these data are considered in the context of broadly contemporary documentary evidence referring to agricultural practices.

A number of direct dates from barley grains on the southern side of the Tibetan Plateau fall within the third millennium BC. The earliest date from northwest India is from Masudspur VII in Haryana (2832–2474 cal. BC; all ages presented at the 95.4% probability range), followed by Burj (2581–2349 cal. BC). In the Ganges, the earliest date is from Damdama (2832–2303 cal. BC), while in south India, the earliest date is from Sannarachamma (1951–1765 cal. BC).

On the northern side of the Tibetan Plateau, the earliest barley dates are from the second millennium BC or later. The earliest date in Central Asia is from Aigyrzhal-2 in Kyrgyzstan (1630–1497 cal. BC), followed by Ojakly in Turkmenistan (1617–1498 cal. BC). A barley grain found at Tasbas in Kazakhstan has been dated to between 1437 and 1233 cal. BC. The earliest date in Xinjiang, west China, is from Sidaogou (975–831 cal. BC). In Gansu province, the earliest date in Heishuiguo has a range of 1880 to 1693 cal. BC, and in central and east China, the earliest date is from Shandong province, with a range of 895 to 751 cal. BC.

The results of the of the Bayesian modelling show a broadly north to south chronological sequence of first appearance dates within South Asia, and a south to north chronological sequence between South and East Asia (see Figs 1–3).

## Discussion: Chronology of spread, and seasons of cultivation

The analysis of extant and new data indicates that the earliest direct radiocarbon dates for barley on the southern side of the Tibetan Plateau are around one millennium older than those on the northern side. Notably, several barley grains from northern India are dated to the third millennium BC, whereas barley grains from Qinghai on the northeastern Tibetan Plateau (the oldest from China) are dated to the early second millennium BC. The equivalent dates from Kazakhstan and Xinjiang range between the late second and first millennia BC, and those from central and eastern China fall in the first millennium BC. Within an overarching trajectory of eastward movement, these results display a south-north chronological offset for barley (Figs 1 and 2), which is in contrast to the equivalent results for wheat, which display a west-east chronological offset (see Fig A and additional text in S1 File).

The earliest direct dates for wheat in India and Kazakhstan fall within the third millennium BC, for Xinjiang, Qinghai and the Hexi Corridor within the early second millennium BC, and from central China within the late second millennium BC [11]. It should be noted that the oldest directly dated wheat is from Zhaojiazhuang (2562–2209 cal. BC) in Shangdong Province in the very east of China [48]. This record may be viewed in the context of an earlier (the third millennium BC) maritime route which has been previously proposed, but is yet to be identified [12].

From this contrast in the pattern of dates we infer that the eastward spreads of wheat and barley did not follow the same initial route. A northern route for wheat is relatively unproblematic, and much has been written about an Inner Asian Mountain Corridor, via the Tianshan and Hexi corridors, which constitutes a likely candidate [11,29]. Elucidation of a distinct and more southerly route for barley (south of the Tibetan Plateau and via the plateau) is possible, but conceptually more challenging. In this context, two areas are important for future study. Along the northern route, remains of naked barley—both grains and chaffs—are documented from Shortughai in Afghanistan, Sarazm in Tajikistan and Begash in Kazakhstan, all from possible third-second millennia BC deposits, but barley has not been directly dated from these sites thus far [14,32,33]. Along the southern route, we are lacking evidence for third millennium BC barley from southern Tibet, which might contextualise the data points in the northeastern Tibetan Plateau. Further investigation in these areas would be of great value.

A contrast between the patterns for wheat and barley may also be discerned in the context of their dispersals into central and eastern China. Between these two dispersals, there appears to have been a considerable time lag. Wheat and barley are both recorded from the northeastern Tibetan Plateau and the Hexi corridor around 2,000 BC. From there, free-threshing wheat moved to central and eastern China around 1,500 BC (notwithstanding a single earlier date from Zhaojiazhuang). However, barley is not recorded in this region until 900 BC.

The arrival of barley in central China is sufficiently late to coincide with some of the earliest Chinese texts. The oldest textual evidence of the Fertile Crescent crops in China comes from oracle bone inscriptions recovered from Anyang, Henan province. These inscriptions were carved on bones and turtle shells and dated to *c*. 1,500–1,000 BC [49]. The words *Lai* and *Mai* (来, 麦) were both used in oracle bone texts to refer to a type of cereal. *Lai* is known to denote wheat [50], but it is unclear whether *Mai* was used to denote wheat alone or wheat and/or barley collectively in the manner of its use in contemporary Chinese. A third character, *Mou* (牟), refers specifically to barley, but it does not appear in the oracle bone inscriptions [50,51]. These references are consistent with the inference from dated grains that wheat cultivation may predate barley cultivation in central and eastern China by several centuries.

Some first millennium BC texts detail agrarian practices in central/eastern China, with frequent references to the planting and harvesting of wheat and barley [51]. From them, we may infer the flexibility in planting and harvesting times of barley (and wheat) in this part of China in the first millennium BC. The different seasons of planting and harvesting referred to in the texts are listed in Table 2, with original texts and translations included in Table A in S1 File. Five out of nine records make reference to wheat and barley harvesting times, and they range between May and September. Three records make reference to different sowing times, two in the autumn one in the spring. It is worthy of a note that the only record of the ‘spring sowing’ occurred in the very late of the first millennium BC. What these texts suggest is the flexibility in growing seasons of barley (and wheat) in the first millennium BC. This pattern resonates with the risk aversion strategy employed by farmers today at the vertical transactions of the edge of the Tibetan Plateau (detailed in the additional text and Figs C and D in S1 File). In Qinghai and Gansu today, as they move to and live at different altitudes, farmers vary the sowing and harvesting times of crops in order to avoid early frosts as they move to and live at different altitudes.

**Table 2:** References to planting and harvesting time of barley/wheat in ancient Chinese texts from the first millennium BC. See Table A S1 File for original texts/translations and more information about the chronology of the texts.

Chronology of the text		Source	Name of crop in the text	Type of crop	Seasonal information in the text	Seasonality in Gregorian calendar and the activity
The thirteenth—twelfth Century BC		甲骨文Oracle bone inscription	麦*Mai*	Likely wheat	正一月The first month	April /May (eating)
The eighth–fifth Century BC		诗经·豳风·七月*Book of Songs*, *Bin Feng*, *Qi Yue*	麦*Mai*	Wheat and/or barley	十月The tenth month	August/September or October/November (harvesting)
The fourth Century BC		左传·隐公三年*Zuo Zhuan*, *Duke Yin of Lu*, *Year Three*	麦*Mai*	Wheat and/or barley	夏四月The fourth month in summer	May (harvesting)
The fourth Century BC		左传·庄公七年*Commentary of Zuo*, *Duke Zhuang of Lu*, *Year Seven*	麦*Mai*	Wheat and/or barley	秋Autumn	June/July (no harvest because of flood)
The fourth Century BC	左传·成公十年*Commentary of Zuo*, *Duke Cheng of Lu*, *Year of Ten*		麦*Mai*	Wheat and/or barley	六月The sixth month	May (deliver the first harvest)
Around the common year	管子·轻重*Guan Zi*, *Qing Zhong*		麦*Mai*	Wheat and/or barley	九月The ninth month	October/November (planting) 20^th^– 22^nd^ June (harvesting)
Early third Century BC	孟子·告子章句上*Mencius*, *Gao Zizhang Ju Shang*		麰麦*Mou Mai*	Barley	日至之时Summer solstice	20^th^–22^nd^ June (harvesting)
Late third Century BC	吕氏春秋·任地*The Annuals of Lu*, *Ren Di*		大麦 *Da Mai*	Barley	孟夏Early Summer	May (harvesting)
Early first Century BC	礼记·月令*Book of Rites*, *Yue Ling*		麦 *Mai*	Wheat and/or barley	仲秋Early autumn	September/October (sowing)
Late first Century BC	氾胜之书*Book of Fan Shengzhi*		宿麦 *Su Mai*	Winter wheat and/or barley	夏至后七十日Seventy days after the summer solstice	September/October (sowing)
Late first Century BC	氾胜之书*Book of Fan Shengzhi*		旋麦 *Xuan Mai*	Spring wheat and/or barley	春冻解After defrost in spring	February/March (sowing)

**Fig 4:**
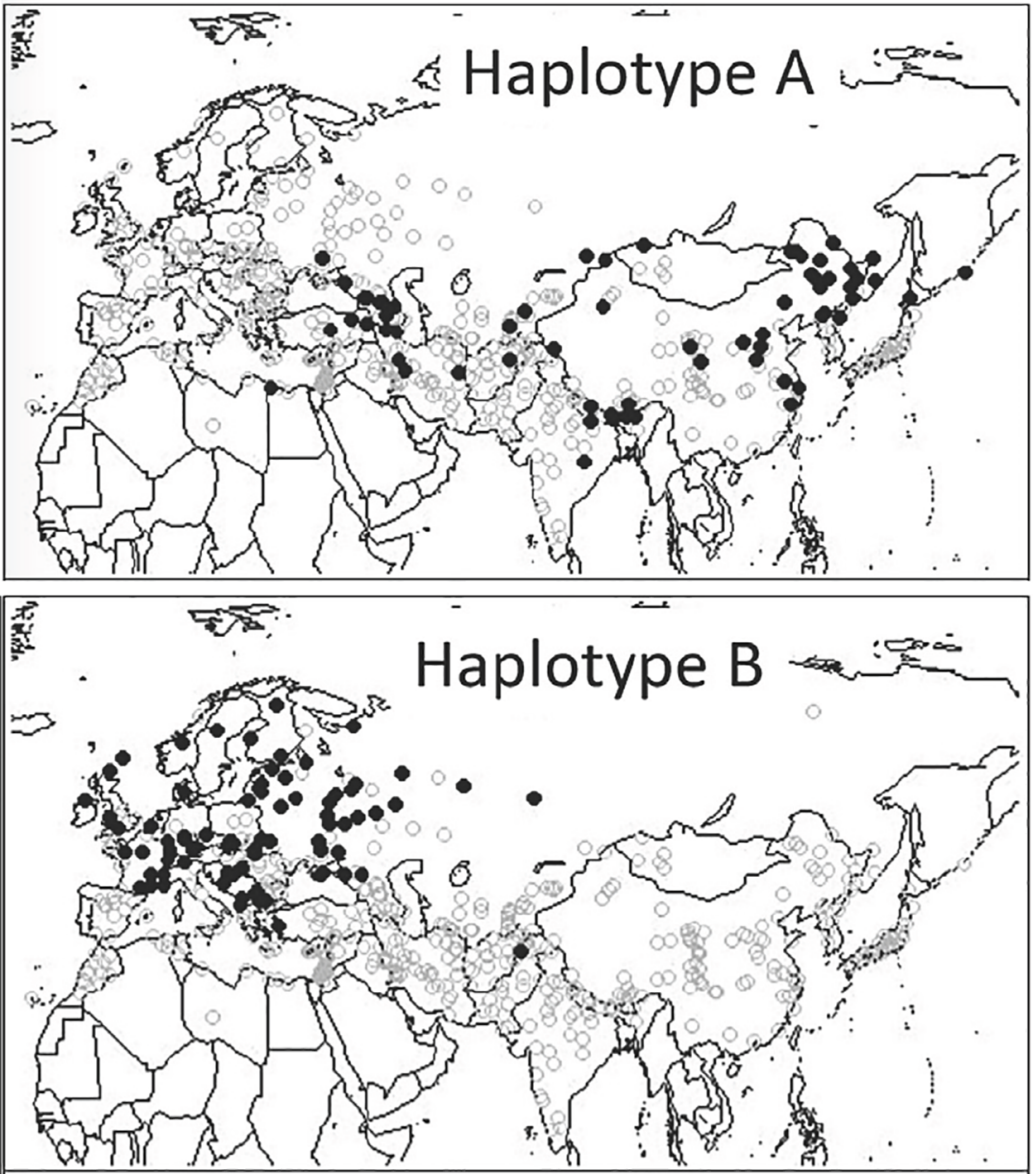
Geographic distributions of the non-responsive haplotypes A and B of the Ppd-H1 gene in extant landrace barley. Re-drawn and modified after Fig 2 in [1].

We may also infer that some of the barley that was being cultivated in this part of China had already acquired mutations in genes involved in flowering time, such as *Ppd-H1*. This inference may be viewed in the context of the observation that in extant landraces cultivated today, distinct *Ppd-H1* haplotypes are differentially distributed across Eurasia [1]. Two of these haplotypes have the non-responsive form of the *Ppd-H1* gene, A and B, where plants do not flower in response to long days. Haplotype B is found almost exclusively in European barley landraces, and their geographical distribution is consistent with adaptation to more northerly latitudes. The geography of the distinct Haplotype A, presenting among Asian landraces, is most simply accounted for eastward dispersals towards both higher altitudes and more northerly latitudes in Central Asia and the Tibetan Plateau (Fig 4). The other six *Ppd-H1* haplotypes display the wild type photoperiod responsive form of *Ppd-H1* and are differentially distributed across Eurasia, with haplotypes C and G common in East Asia. From these distributions we may infer that barley both with photoperiod responsive and with non-responsive alleles of the *Ppd-H1* gene have been successfully cultivated in central/eastern China in the first millennium BC.

## Conclusions

In this paper, we demonstrate that, before reaching central/eastern China in the first millennium BC, barley had been cultivated in a range of markedly contrasting environments, consistent with its distinctive ecological versatility as a taxon. Fig 1 shows barley cultivation ranging from arid temperate Central Asia to semi-tropical south India during the second millennium BC–a latitudinal range greater than 40 degrees. It also ranges from the lowland Ganges to highland Tibetan Plateau–an altitudinal span exceeding 3,500 meters. These contrasting situations provided the context for adaptive changes in flowering time genes, pre-adapting them to cropping systems in the central plains of China that favoured multiple forms. The data presented here allow for two principal inferences, firstly relating to different eastward dispersals of western cereals, and secondly to patterns in the associated seasonalities of those cereals.

The first inference is that the eastern dispersals of wheat and barley are distinct in both space and time. Previous discussions have often focused on the northern edge of the Tibetan Plateau, but we call for attention to the possibility of a southern route that may better accord with the radiocarbon dating evidence.

The second inference concerns the topographical routes of those dispersals, and the associated adaptive challenges. Barley arrives in the Central Plains later than wheat, bringing with it a degree of genetic diversity in relation to flowering time responses. This may be inferred both from the genetic diversity of extant landraces from the region, and from contemporary texts documenting a diversity of sowing and harvesting times for barley. Such diversity may in turn reflect preadaptation of barley varieties along the eastward route to seasonal challenges, either at northerly latitudes or higher altitudes. The west-east disjunct in non-responsive haplotypes of flowering time gene (A and B in *Ppd-H1*) is more easily explained by a prominence of the latter pathway, following higher altitude. This in turn draws attention to both the known ecological versatility of barley in comparison to wheat.

## Supporting information

S1 File (including Table A and Figs A-C).
(DOCX)
