# Correction: Journey to the east: Diverse routes and variable flowering times for wheat and barley *en route* to prehistoric China

**DOI:** 10.1371/journal.pone.0209518

**Published:** 2018-12-13

**Authors:** Xinyi Liu, Diane L. Lister, Zhijun Zhao, Cameron A. Petrie, Xiongsheng Zeng, Penelope J. Jones, Richard A. Staff, Anil K. Pokharia, Jennifer Bates, Ravindra N. Singh, Steven A. Weber, Giedre Motuzaite Matuzeviciute, Guanghui Dong, Haiming Li, Hongliang Lü, Hongen Jiang, Jianxin Wang, Jian Ma, Duo Tian, Guiyun Jin, Liping Zhou, Xiaohong Wu, Martin K. Jones

The following information is missing from the Funding section: The contribution made by C. A. Petrie was supported by funding from European Research Council (ERC) under the European Union’s Horizon 2020 research and innovation programme (no 648609).
