# Supplementary material for: Journey to the east: Diverse routes and variable flowering times for wheat and barley en route to prehistoric China
Source: PLoS One. 2017 Nov 2;12(11):e0187405. doi: 10.1371/journal.pone.0187405 (PMC5667820; doi:10.1371/journal.pone.0187405)
Supplement: S1 File — (including Table A and Figs A-C). (DOCX) [file pone.0187405.s001.docx]

**S1 File: Supporting information for**

The origin of eastern spring barley: diverse routes and ecological challenges for wheat and barley *en route* to prehistoric China

Xinyi Liu, Diane L. Lister, Zhijun Zhao, Cameron A. Petrie, Xiongsheng Zeng, Penelope J. Jones, Richard A. Staff, Anil K. Pokharia, Jennifer Bates, Ravindra N. Singh, Steven A. Weber, Giedre Motuzaite Matuzeviciute, Guanghui Dong, Haiming Li, Hongliang Lü, Hongen Jiang, Jianxin Wang, Jian Ma, Duo Tian, Guiyun Jin, Liping Zhou, Xiaohong Wu and Martin K. Jones

e-mail: liuxinyi@wustl.edu

**Historical textual information**

Early Chinese texts in the second and first millennium BC notably document a wide range of variation in planting and harvesting times of wheat and barley. Table 2 in the main text lists the different seasons of planting and harvesting appearing in these texts. Table A includes the source of the text with English translation. Wheat and barley harvesting times range between May and September. For example, in *Shi Jing* (the Book of Songs), a document written in the early centuries of the first millennium BC, wheat and/or barley was harvested in autumn (likely August-September) together with broomcorn millet [[1](#_ENREF_1)]. A third century BC text, *Lu Shi Chun Qiu* (Lü’s Annals), documents barley being harvested in May [[2](#_ENREF_2)]. In *Meng Zi* (Mencius), written in the latter part of the first millennium BC, barley was harvested upon the summer solstice [[3](#_ENREF_3)]. In *Fan Shengzhi Shu* (The Book of Fan Shengzhi), the first comprehensive agricultural book in Western Han (202 BC – 8 AD), the planting times of spring and winter wheat and barley are recorded [[4](#_ENREF_4), [5](#_ENREF_5)]. Drawing from these documents, we can infer that the cultivation of barley (and wheat) in the first millennium BC featured both spring and winter varieties.

**The Eastern Spread of Wheat**

In order to understand the eastward expansion of another Near Eastern crop, free threshing wheat, we previously obtained direct radiocarbon measurements from 51 charred wheat grains^4^. From these dates, together with previously published data, we could distinguish separate sequences along the north and south of the Tibetan Plateau. In the north, the sequence runs from the eastern range of the Inner Asian Mountain Corridor through the Tianshan Mountains and the Hexi Corridor. It then extends to the middle and lower reaches of the Yellow River. In the south, though on the basis of fewer data, the sequence runs from northwest India through to south India and the Ganges region. Thus, the origin of free-threshing wheat in Tibet could be from either the northern or the southern corridor. Fig A shows the location of archaeological sites from which wheat grains were dated. These results show a west to east chronological sequence in Central and East Asia, and a north to south sequence in South Asia for wheat [[6](#_ENREF_6)].

The adoption of wheat along the northern corridor was initially rapid in northwestern China. This rapid pace was then interrupted in eastern Gansu. The pace then recovered in central China after 1650 cal. BC. The changing pace of this eastward expansion of wheat has been considered in the context of environmental and culinary differences between monsoonal and non-monsoonal regions of China [[6](#_ENREF_6)].

**The Vertical Landscapes of Barley Cultivation in Qinghai**

Many of the sites with barley dated to the second millennium BC occur along vertical transects on the northeast margins of the Tibetan Plateau. For example, three Qinghai sites provide direct dates for barley prior to 2000 BC, constituting the oldest records of barley in China. They all appear at elevations over 2000 m.a.s.l. Another 19 sites report direct dates for barley in this region prior to 1000 BC. These later sites are located along a vertical transect on the northwestern edge of the Tibetan Plateau (see Fig B), ranging from 1473 to 3341 m.a.s.l. Four of these sites lie below 2000 m.a.s.l., ten between 2000-2500 m.a.s.l., five between 2500-3000 m.a.s.l., and three above 3000 m.a.s.l. [[7](#_ENREF_7)].

The highly variable temperature and water availability in high altitude environments can generate high levels of genetic and phenotypic diversity [[8-10](#_ENREF_8)], which includes variations in crop seasonality. On the Tibetan Plateau, flexibility in sowing and harvesting times has always been a crucial risk aversion strategy employed by farmers, both in the past and present [[11-13](#_ENREF_11)]. In Qinghai and Gansu today, farmers vary the sowing and harvesting times of crops in order to avoid early frosts as they move to and live at different altitudes (see Fig C).

**Recent Study of the *Ppd-H1* gene in barley landraces**

A recent study extended the analysis of the *Ppd-H1* gene beyond Europe to include 510 well-provenanced barley landraces from across the Eurasian continent and North Africa [15]. This study identified a number of *Ppd-H1* haplotypes with distinct phylogeographies, two of which, A and B, possess the form of the SNP that turns off flowering in response to long days and another six of which possess the wild type form of *Ppd-H1*, where flowering is initiated by long days (C to H). Each haplotype has a distinctive distribution across Eurasia, which may reflect different facets of the global spread of barley. The non-responsive haplotypes A and B have a clear east-west distribution that suggests independent spread of each non-responsive haplotype (see Fig 4 in the main text), with haplotype A being broadly distributed across Eurasia, including China and India, and haplotype B being mostly restricted to Europe.

Turning to the responsive *Ppd-H1* haplotypes, C is widespread across the Tibetan Plateau and is also present in parts of Central Asia and Mongolia. We may infer that these responsive barleys found at high altitudes on the Tibetan Plateau, including extant accessions in Qinghai where the earliest barley in China has been recorded, have adapted in other ways to the extremes of a high altitude environment, for example, by acquiring frost tolerance [[14](#_ENREF_14)]. The responsive haplotype G is found in Iran and Eastern China, and is the most common haplotype in Japan; and has raised the question about possible connections between those regions [[15](#_ENREF_15)]. A possible scenario is that both non-responsive and responsive barleys moved from the high altitudes of the Tibetan Plateau, to the temperate lowlands in central/eastern China in the first millennium BC, and were incorporated into early farming practices, which, as we have discussed above from textual evidence, shows barley with different seasonalities being grown.

**References**

1. Zhou Y, editor. Zhongguo Lishi Wenxuan [Selections from Chinese Ancient Text]. Beijing: Zhonghua Shuju [Chung Hwa Book Co.]; 1961.

2. Lü [Qin Dynasty] B, editor. Lüshi Chunqiu Xin Jiaoyi [New Translation of Lü's Annuals]. Shanghai: Shanghai Guji Chubanshe [Shanghai Classic Publishing House]; 2002.

3. Wang [Song Dynasty] Y, editor. Mencius. Shanghai: Shanghai Guji Chubanshe [Shanghai Classic Publishing House]; 2015.

4. Jia [Hou Wei Dynasty] S, editor. Qimin Yaoshu [Essential techniques for the welfare of the poeple]. Beijing: Nongye Chubanshe [Agricultural Publishing House]; 1982.

5. Ban [Han Dynasty] G, editor. Han Shu [Book of Han]. Beijing: Zhonghua Shuju [Chung Hwa Book Co.]; 1962.

6. Liu X, Lister DL, Zhao Z-Z, Staff RA, Jones PJ, Zhou L-P, et al. The virtues of small grain size: Potential pathways to a distinguishing feature of Asian wheats. Quaternary International. 2016;426:107-9.

7. Chen F-H, Dong F-H, Zhang D-J, Liu X-Y, Jia X, An C-B, et al. Agriculture facilitated permanent human occupation of the Tibetan Plateau after 3600BP. Science. 2015;347(6219):248-50.

8. Engels JMM. Genetic diversity in Ethiopian barley in relation to altitude. Genetic Resources and Crop Evolution. 1994;41:67-73.

9. Hadado TT, Rau D, Bitocchi E, Papa R. Adaptation and diversity along an altitudinal gradient in Ethiopian barley (Hordeum vulgare L.) landraces revealed by molecular analysis. BMC Plant Biology. 2010;10(121):DOI: 10.1186/471-2229-10-121.

10. Abebe TD, Bauer AM, J. L. Morphological diversity of Ethiopian barley (Hordeum vulgare L.) in relation to geographic regions and altitudes. Hereditas. 2010;147:154-64.

11. QHSKJT [Bureau of Science and Technology of Qinghai]. Chun Xiaomai Zaipei Jishu [The Cultivation of Spring Type of Wheat]. Xining: Qinghai Renmin Chubanshe [People's Publishing House of Qinghai]; 2007.

12. Ma Q, Liu H, Chen L, Yang L. Gansu Sheng Zaipei Damei Shengtai Qu de Chubu Huafen [Ecological zones of barley cultivation in Gansu Province]. Gansu Nongye Keji [Gansu Agricultural Sciences]. 1989;1:1-3.

13. Yang F. Qinghai Dongbu Huangtu Yaoyuan Hanzuo Tudi Liyong de Tantao [The utilization of soil in the loess agricultural zone in eastern Qinghai province]. Shuitu Baochi Yingyong Jishu [Water and Soil Conservation]. 1991;1:63-4.

14. Galiba G, Vágújfalvi A, Li C, Soltész A, Dubcovsky J. Regulatory genes involved in the determination of frost tolerance in temperate cereals. Plant Science. 2009;176(1):12-9.

15. Jones H, Lister DL, Cai D-W, Kneale CJ, Cockram J, Peña-Chocarro L, et al. The trans-Eurasian crop exchange in prehistory: discerning pathways from barley phylogeography. Quaternary International. 2016;426:26-32.

16. Peng B. Jiaguwen Nongye Ziliao Kaobian yu Yanjiu [Investigating the Agricultral Texts in Orical Bone Inscriptions]. Changchun: Jilin Wenshi Chubanshe [Jilin Publishing House for Literature and History]; 1997.

17. Chang Y. Yinshang Lifa Yanjiu [Studies of Calendars in Yinshang Period]. Changchun: Jilin Wenshi Chubanshe [Jilin Publishing House for Literature and History]; 2998. 407 p.

18. Chang Y. Yinshang Lifa Yanjiu [Studies of Calendars in Yinshang Period]. Changchun: Jilin Wenshi Chubanshe [Jilin Publishing House for Literature and History]; 1998.

19. Zhou Y, editor. Zhongguo Lishi Wenxuan [Selections from Chinese Ancient Text]. Beijing: Zhongguo Lishi Wenxuan [Selections from Chinese Ancient Text ]; 1961.

20. Hong [Qingh Dynasty] L, editor. Chunqiu Zuozhuan - Zhuan - Yingong - Sannian [Commentary of Zuo, Book I, Duke Yin, Year Three]. Beijing: Zhonghua Shuju [Chung Hwa Book Co.]; 1987.

21. Ma B. Guanzi Qingzhong Pian Xinquan [New Interpretation of Qingzhong, a Chapter in Guan Zi]. Beijing: Zhonghua Shuju [Chung Hwa Book Co.]; 1979. 3 p.

22. Co.] ZSCHB. Zhonghua Shuju [Chung Hwa Book Co.]. Shanghai: Zhonghua Shuju [Chung Hwa Book Co.]; 2009. 11 p.

**Table A** Texts from the first millennium BC that mention planting and harvesting times of barley/wheat.

| Text and translation | Source | Date information | Reference |
| --- | --- | --- | --- |
| 正一月曰食麦Eat wheat in the first month. | 甲骨文Oracle bone inscription | Prior to 1169 BC. The piece of bone was recovered from the second phase of the Shang deposit at Anyang. It is generally believed that this period was under the rule of King Zugeng and Zujia. | [[16-18](#_ENREF_16)] |
| 九月筑场圃，十月纳禾稼，黍稷重穋，禾麻菽麦。In the ninth month threshing sites are prepared; In the tenth month crops of all kinds come in baled: Broomcorn millet, grains ripen early or late; Also we have foxtail millet, hemp, beans and wheat/barley. [trans. James Legge] | 诗经·豳风·七月*Book of Songs, Bin Feng, Qi Yue* | The book of Songs was probably written in the Spring and Autumn Period (770-476 BC). | [[19](#_ENREF_19)] |
| 夏四月，郑祭足帅师取温之麦。In the fourth month in the summer, Zheng led a force and carried away the wheat/barley of Wen. [trans. James Legge] | 左传·隐公三年*Commentary of Zuo, Duke Yin of Lu, Year Three* | *Zuo Zhuan* was written in the middle Warring States Period (the fourth Century BC). This chapter, Duke Yin, Year Three, refers to the year of 720 BC. | [[20](#_ENREF_20)] |
| 秋无麦苗，不害嘉谷也。In the Autumn, there is no harvest of the wheat/barley, other grains also failed. | 左传·庄公七年*Commentary of Zuo, Duke Zhuang of Lu, Year Seven* | *Commentary of Zuo* was written in the middle Warring States Period (the fourth Century BC). This chapter, Duke Zhuang, Year Seven, refers to the year of 687 BC. | [[20](#_ENREF_20)] |
| 六月，丙午，晋侯欲麦，使甸人献麦。In the sixth month, on day Bing Wu, the Duke Jin requested the (first harvest) of wheat/barley, (officials) were sent to Dian for wheat/barley. | 左传·成公十年*Commentary of Zuo, Duke Cheng of Lu, Year of Ten* | *Commentary of Zuo* was written in the middle Warring States Period (the fourth Century BC). This chapter, Duke Cheng, Year Ten, refers to the year of 581 BC. | [[20](#_ENREF_20)] |
| 乃请以令使九月种麦, 日至日获,则时雨未下, 而利农事矣。Please give the order to sow wheat/barley in the ninth month, harvest the grains on the summer solstice. This will promise a good harvest since it is not the rainy season when harvesting. | 管子·轻重*Guan Zi, Qing Zhong* | The book, *Guan Zi*, was written around the common year. The text refers to a conversation in 645 BC. | [[21](#_ENREF_21)] |
| 今夫麰麦，播种而耘之，其地同，树之时又同，浡然 而生，至于日至之時，皆熟矣。Now if you plant wheat and barley and cover them, and the soil is the same, and they are planted at the same time, they will all grow strongly. And when the time is right around the summer solstice, they will all be ripe. Even though there are differences, it is because of differences in soil fertility, the nourishment from rain, or the amount of care and cultivation given by the farmers. [trans. A. C. Muller] | 孟子·告子章句上*Mencius, Gao Zizhang Ju Shang* | Prior to 289 BC. It is generally believed that *Mencius* was completed by Mencius, who died in 289 BC, and his students. | [[3](#_ENREF_3)] |
| 孟夏之昔， 杀三叶而获大麦。Before the fourth month in summer, when three leaves die away, is the time to harvest barley. [three leaves refer to three cruciferous plants, shepherd’s purse (*Capsella bursa-pastoris*), Rorippa montana and field pennycress (*Thlaspi arvense*) ] | 吕氏春秋·任地The Annuals of Lü*, Ren Di* | Prior to 237 BC (The tenth year of Qin) | [[2](#_ENREF_2)] |
| 仲秋之月……乃命有司，趣民收斂，務畜菜，多積聚。乃劝人种麦，无或失时，刑罪无疑。In the eighth month…… orders are given to the proper officers to be urgent with the people, and (to finish) receiving their contributions and storing them. They should do their best to accumulate (large) stores of vegetables and other things. They should (also) stimulate the wheat/barley-sowing. (The husbandmen) should not be allowed to miss the proper time for the operation. Any who do so shall be punished without fail. [trans. James Legge] | 礼记·月令*Book of Rites – Yue Ling* | Prior to 51 BC (The third year of Ganlu, Empire Xuan of Han) | [[22](#_ENREF_22)] |
| 夏至后七十日，可以种宿麦。Seventy days after the summer solstice, winter wheat/barley may be sown. | 氾胜之书*Book of Fan Shengzhi* | 33-7 BC | [[4](#_ENREF_4), [5](#_ENREF_5)] |
| 春冻解，耕和土，种旋麦。When it defrosts in spring, this is the time to plow the soil with the rising temperature, and plant spring wheat/barley. | 氾胜之书*Book of Fan Sheng* | 33-7 BC | [[4](#_ENREF_4), [5](#_ENREF_5)] |


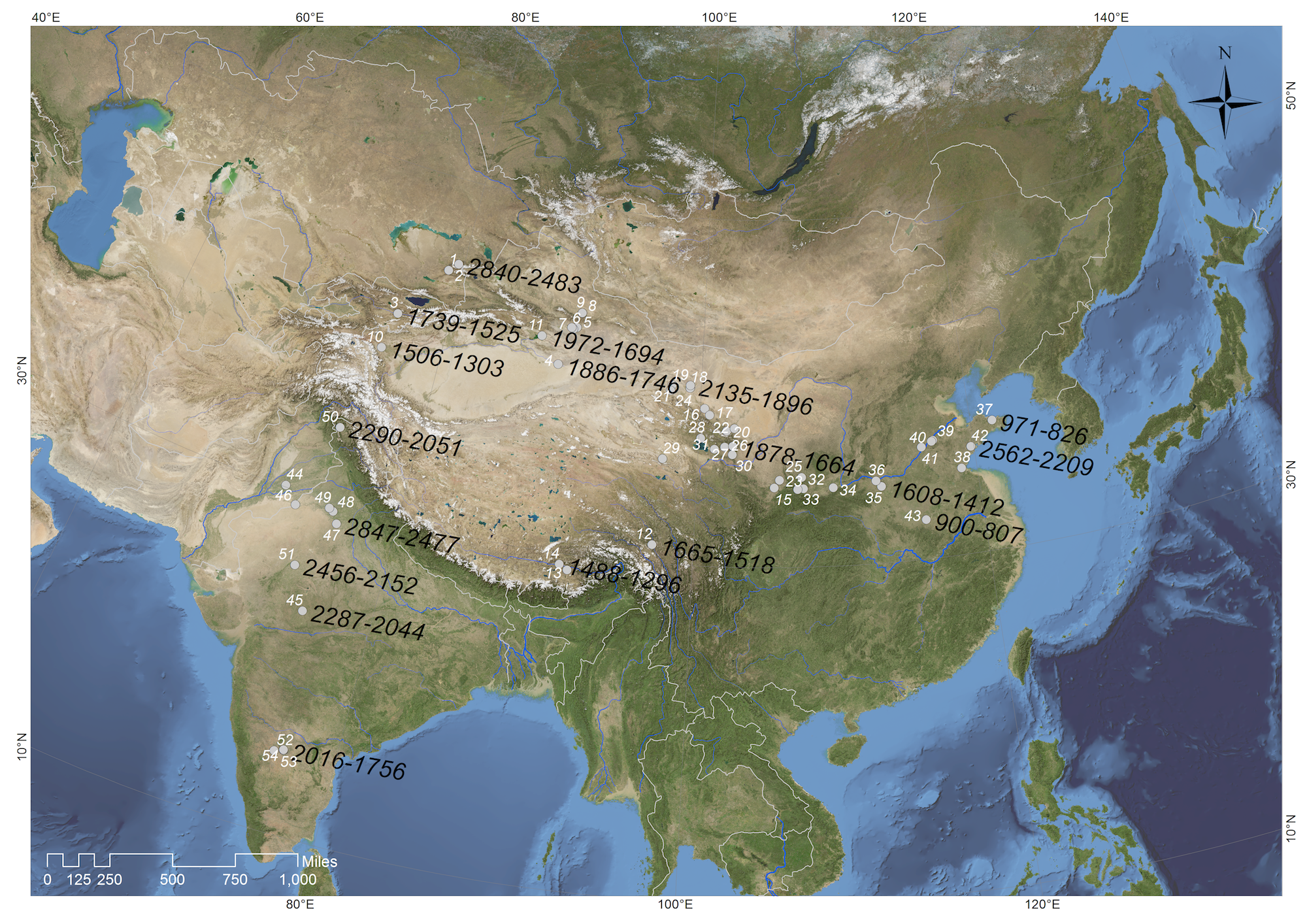


**Figure A** Locations of sites where archaeobotanical wheat grains have been dated and published. The oldest individually dated grains of free-threshing wheat from each region are indicated. (Re-dawn and modified after Figure 1 in [[6](#_ENREF_6)]). 1. Begash, 2. Tasbas 1, 3. Aigyrzhal-2, 4. Gumugou, 5. Shengjindian, 6. Yanghai, 7. Astana, 8. Sidaogou, 9. Xicaozi, 10. Wupaer, 11. Xintala, 12. Karou, 13. Bangga, 14. Changgougou, 15. Xishanping, 16. Heishuiguo, 17. Donghuishan, 18. Ganggangwa, 19. Huoshiliang, 20. Mozuizi, 21. Shaguoliang, 22. Huangniangniangtai, 23. Dadiwan, 24. Huoshaogou, 25. Qiaocun, 26. Fengtai, 27. Jinchankou, 28. Aiqingya, 29. Xiariyamakebu, 30. Shuangerdongping, 31. Longshan, 32. Zhouyuan, 33. Shangguancun, 34. Nansha, 35. Wangchenggang, 36. Yanshishangcheng, 37. Zhaogezhuang, 38. Dongpan, 39. Daxinzhuang, 40. Liujiazhuang, 41. Jiaochangpu, 42. Zhaojiazhuang, 43. Yantai, 44. Harappa, 45. Khirsara, 46. 4-MSR/Binjore, 47. Tigrana, 48. Kunal, 49. Banawali, 50. Kanispur, 51. Ojiyana, 51. Pikliha, 53. Sannarachamma, 54. Hiregudda.

**
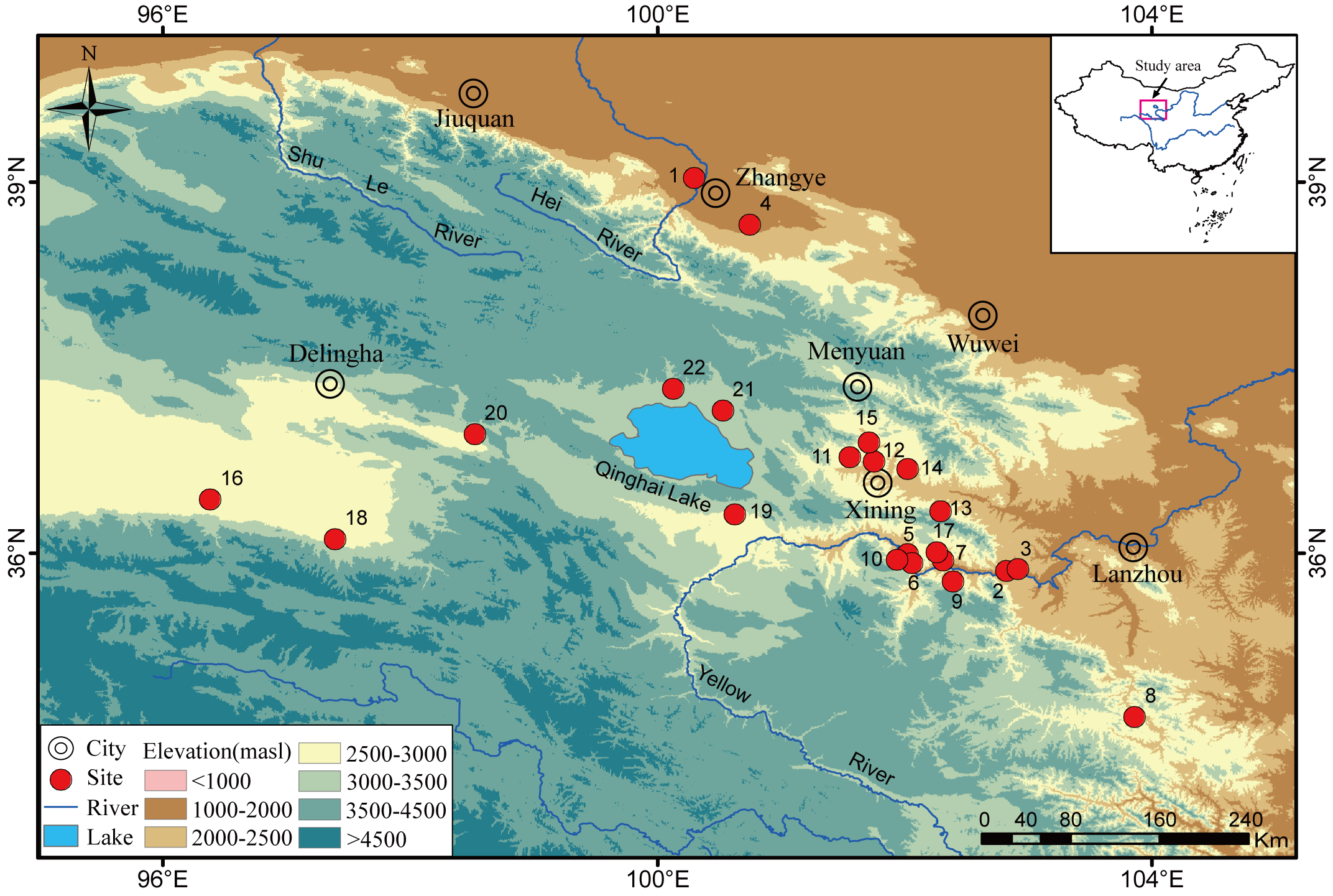
**

**Figure B** The 22 sites from Qinghai and Gansu with directly radiocarbon dated barley yielding ages prior to 1000 BC. These sites are located along a vertical transect on the northwestern edge of the Tibetan Plateau, ranging from 1473 to 3277 m.a.s.l. Four of the sites lie below 2000 m.a.s.l. (1. Heishuiguo, 2. Erfang, 3. Wenjia, 4. Donghuishan), ten between 2000-2500 m.a.s.l. (5. Dongfengxinan, 6. Tuanjie, 7. Gongshijia, 8. Mogou, 9. Jiaoridang, 10. Luowalinchang, 11. Xiasunjiazhai, 12. Changning, 13. Huidui, 14. Weijiabao), five between 2500-3000 m.a.s.l. (15. Kalashishuwan, 16. Talitaliha, 17. Bayan, 18. Tawendaliha, 19. Qiezha), and three above 3000 m.a.s.l. (20. Hongshanzuinanpo, 21. Caodalianhuxi, 22. Lagalamaerma).

**Figure C** Current day sowing and harvesting times for spring barley in Qinghai and Gansu. Note that the times that farmers plant and harvest barley show considerable variability [[11-13](#_ENREF_11)].
